# Telemedicine Use and Quality of Opioid Use Disorder Treatment in the US During the COVID-19 Pandemic

**DOI:** 10.1001/jamanetworkopen.2022.52381

**Published:** 2023-01-24

**Authors:** Ruth Hailu, Ateev Mehrotra, Haiden A. Huskamp, Alisa B. Busch, Michael L. Barnett

**Affiliations:** 1 Department of Health Care Policy, Harvard Medical School, Boston, Massachusetts; 2 Division of General Medicine, Beth Israel Deaconess Medical Center, Boston, Massachusetts; 3 McLean Hospital, Belmont, Massachusetts; 4 Department of Health Policy and Management, Harvard T.H. Chan School of Public Health, Boston, Massachusetts; 5 Division of General Internal Medicine and Primary Care, Department of Medicine, Brigham and Women’s Hospital, Boston, Massachusetts

## Abstract

**Importance:**

Little is known about the potential implications of the rapid transition to telehealth during the COVID-19 pandemic for treatment of opioid use disorder (OUD).

**Objective:**

To examine the association between telemedicine adoption during the COVID-19 pandemic and indicators of OUD treatment quality.

**Design, Setting, and Participants:**

This cohort study analyzed deidentified administrative claims data from OptumLabs Data Warehouse. Claims for telemedicine visits were included for both the prepandemic period (March 14, 2019, to March 13, 2020) and pandemic period (March 14, 2020, to March 13, 2021). Patients with OUD and continuous enrollment in either commercial insurance or Medicare Advantage plans were included. Clinicians who provided office-based OUD care were included and categorized into low, medium, or high telemedicine use groups. Patients were attributed to the clinician (and corresponding telemedicine use group) from whom they received a plurality of OUD visits.

**Main Outcomes and Measures:**

The 4 outcomes were all outpatient visits, OUD visits (in person vs telemedicine) within 90 days of an index visit, medications for OUD (MOUD) prescribing, and OUD-related clinical events (including drug overdose, inpatient detoxification and rehabilitation center stay, or injection drug use–related infections).

**Results:**

The analysis included 11 801 patients (mean [SD] age, 53.9 [15.7] years; 5902 males [50.0%]) who were treated by 1768 clinicians. Clinicians with low vs high telemedicine use conducted a mean (SD) of 2.1% (2.5%) vs 69.5% (18.6%) of their office visits virtually in the pandemic period. While telemedicine use for OUD increased significantly from the prepandemic to pandemic periods, total OUD visit volume (in person plus telemedicine) per patient episode remained stable among both high (2.6 to 2.7 visits per patient episode) and low (3.1 to 3.3 visits per patient episode) telemedicine use groups. In adjusted analyses comparing the prepandemic with pandemic periods, there was no differential change in MOUD initiation (adjusted odds ratio [OR], 1.00; 95% CI, 0.84-1.19), MOUD days’ supply (differential change in days’ supply, –0.27; 95% CI, –1.84 to 1.30), or OUD-related clinical events (adjusted OR, 1.01; 95% CI, 0.73-1.24) among patients who were treated by clinicians in low vs high telemedicine use groups.

**Conclusions and Relevance:**

Results of this study revealed that clinical outcomes were similar among patients who were treated by clinicians with high and low telemedicine use during the COVID-19 pandemic, suggesting that telemedicine is a comparable alternative to in-person OUD care. There was no evidence that telemedicine was associated with increased access to or improved quality of OUD treatment.

## Key Points

**Question:**

Is clinician telemedicine use during the COVID-19 pandemic associated with differences in care for opioid use disorder (OUD)?

**Findings:**

In this cohort study of 11 801 patients with OUD with commercial insurance or Medicare Advantage coverage, there were no significant differences in visit frequency, initiation of medications for OUD, or OUD-related adverse outcomes between those who were treated by clinicians with high vs low telemedicine use across the prepandemic and pandemic periods.

**Meaning:**

This study found no evidence that telemedicine was unsafe or overused or was associated with increased access to or improved quality of OUD care, suggesting that telemedicine may be a comparable alternative to in-person OUD care.

## Teaser

This cohort study assesses the level of telehealth implementation and its outcomes among office-based clinicians who provide outpatient opioid use disorder care.

## Introduction

Overdose deaths related to opioid use disorder (OUD) have increased rapidly in the past decade from 21 000 in 2010 to over 100 000 in 2021.^1,2^ However, access to OUD treatment has remained limited. Medications for opioid use disorder (MOUD), which include methadone, buprenorphine, and long-acting injectable naltrexone, are considered the most effective treatments available for OUD.^3,4,5,6,7,8,9,10^ However, in 2019, the National Survey on Drug Use and Health found that just 27.8% of individuals who needed OUD treatment received MOUD.^11^ For decades, there have been long-standing barriers to OUD care, including clinician shortages, stigma, cost, and transportation challenges.^12^ The situation may have worsened with the social isolation and infection control interventions during the COVID-19 pandemic, which appear to have played a role in the increased demand for OUD treatment and reduced access to in-person care.^13,14^

Telemedicine has been proposed as a potential solution to reduced OUD treatment access, but there was little telemedicine use prior to 2020.^15^ During the COVID-19 pandemic, federal and state regulatory changes and expanded reimbursement for telemedicine services facilitated substantial increases in telemedicine use.^16^ For patients with OUD, these regulatory changes removed the requirement of the Ryan Haight Act^17^ to meet with a clinician in person before initiating MOUD.^18,19^ In preliminary qualitative and survey research, OUD clinicians reported that telemedicine has led to increased access to buprenorphine and higher rates of MOUD initiation,^20,21,22,23^ in part by removing transportation barriers and relieving the burdens of those with competing demands, such as child care and work.^24,25^ While telemedicine for OUD may provide these benefits, there are limited national, empirical studies on the benefits or drawbacks of this shift in care delivery.^26,27^ More evidence is needed to inform the ongoing debate about regulations and payment for OUD treatment after the COVID-19 public health emergency ends.^28^

The shift in care delivery during the COVID-19 pandemic toward widespread use of telemedicine provides an opportunity to address this knowledge gap. In this cohort study, we used a national database of commercially insured individuals to examine the association between telemedicine adoption during the COVID-19 pandemic and indicators of OUD treatment quality. We used a difference-in-differences methodologic approach, which reduces bias due to nonrandom patient selection into intervention or control groups. Specifically, we compared patients who received OUD treatment from clinicians with high or medium vs low telemedicine use in the pandemic period, adjusting for the outcomes experienced by patients treated by these clinicians during the prepandemic period.

## Methods

### Data Sources

For this longitudinal retrospective cohort study, we used deidentified claims data from the OptumLabs Data Warehouse, which contains a national data set of medical claims and enrollment records for individuals with commercial insurance or Medicare Advantage coverage that is linked to county-level characteristics from the US Census Bureau. This study was approved by the institutional review board at Harvard Medical School, which waived the informed consent requirement because the study was non–human participant review. We followed the Strengthening the Reporting of Observational Studies in Epidemiology (STROBE) reporting guideline.

We included claims for telemedicine visits from March 14, 2019, to March 13, 2021, allowing for 1 year before and 1 year after the start of the US declaration of the COVID-19 Public Health Emergency. Loosening of telemedicine restrictions by the Centers for Medicare & Medicaid Services happened soon after the public health emergency declaration.^16^ March 14, 2019, to March 13, 2020, was labeled as the prepandemic period, and March 14, 2020, to March 13, 2021, was labeled as the pandemic period.

### Clinician Sample

Clinicians functioned as the unit of treatment assignment. The study was limited to clinicians who were most likely to be office-based (ie, nonopioid treatment program) MOUD prescribers: primary care physicians, psychiatrists, nurse practitioners, anesthesiologists (representing pain medicine specialists), rehabilitation medicine clinicians, neurologists, pediatricians, and obstetricians and gynecologists. We included prescription fills for all possible MOUD in addition to claims for facility-administered medications (eTable 1 in Supplement 1). We defined a MOUD prescriber as a clinician with 1 or more MOUD claims in both the prepandemic and pandemic periods. We focused on buprenorphine (long-acting injectable or oral preparations with naloxone) and naltrexone because they are both available via typical office-based practice, while methadone for OUD care can be dispensed only through opioid treatment programs.

### Defining OUD Visits and Telemedicine Exposure

The study sample was composed of patients with OUD with outpatient episodes of care. We defined outpatient visits using Healthcare Common Procedure Coding System (HCPCS) codes that are specific to clinician offices (eTable 2 in Supplement 1), which excluded, for example, emergency departments, hospital inpatient units, nursing homes, and dialysis facilities. We identified individuals with OUD if they had (1) at least 2 outpatient claims with an *International Statistical Classification of Diseases and Related Health Problems, Tenth Revision* (*ICD-10*) code for OUD (F11.1, F11.2, or F11.9) in any diagnosis field, (2) at least 1 inpatient claim and at least 1 outpatient claim with an *ICD-10* code for OUD, or (3) at least 1 inpatient or outpatient claim with an *ICD-10* code for OUD and at least 1 claim with a confirmatory event (opioid overdose; hepatitis C, an infection potentially secondary to injection drug use; or inpatient detoxification or rehabilitation treatment [definitions are provided in eTable 3 in Supplement 1]) within 90 days before or after the claim with an OUD diagnosis.

Among individuals meeting any of these criteria, the earliest observed visit for OUD after a 90-day clean period (no claims for OUD care, MOUD pharmacy, or HCPCS codes) was considered to be the index OUD visit. Treatment episodes for OUD were defined by all claims occurring within 90 days after the index visit. The same patient could have episodes of care in both the prepandemic and pandemic periods, but we included just 1 episode of care per period. For each episode, patients were attributed to the clinician from whom they received a plurality of their OUD visits during that episode. Patients in the sample were also required to have continuous enrollment in medical and pharmacy benefit for at least 90 days before and after the episode index visit. This requirement allowed us to observe their OUD care use, which was necessary to define an index episode after a clean period and to define use that occurred in the 90-day OUD episodes. The full flow chart of the cohort study design is provided in the eFigure in Supplement 1.

We categorized clinicians according to their proportion of telemedicine use during the pandemic period. Telemedicine visits were identified through modifiers GT, GQ, or 95 on eligible outpatient services or *Current Procedural Terminology* codes 99441 to 99443. Clinicians were then separated into tertiles (low, medium, and high telemedicine use) based on the proportion of all outpatient visits (OUD and non-OUD) conducted by each clinician via telemedicine. We defined clinician telemedicine use by measuring telemedicine use for all outpatient visits (ie, not limited to OUD, using the same HCPCS codes [eTable 2 in Supplement 1]) to avoid potential misclassification due to small sample sizes of OUD visits among clinicians. Telemedicine use across all outpatient visits was highly correlated with telemedicine use limited to OUD visits among clinicians (Pearson ρ = 0.71). Patients with OUD were assigned to the telemedicine use group (high, medium, or low) of their assigned clinician. We used clinician telemedicine group as the key exposure variable, as opposed to comparing in-person with telemedicine care at the visit level, to avoid selection bias by indication due to a clinician using telemedicine for specific reasons within their own patient population.

### Study Outcomes

We examined 4 outcomes: all outpatient visits, OUD visits, MOUD prescribing, and OUD-related clinical events. For outpatient visits, we captured total, in-person, and telemedicine visit volumes. We then captured total, in-person, and telemedicine OUD visits within 90 days of an index visit. For MOUD prescribing, consistent with a previous study,^7^ we defined 2 measures of MOUD initiation: (1) the proportion of patients with OUD with initiation within 90 days of the index visit, and (2) the proportion of patients with OUD with initiation within 14 days of the index visit. Retention of MOUD was defined as at least 1 additional MOUD fill within 30 to 90 days of initiation among those with initiation within 14 days of the index visit. We calculated the mean days’ supply of MOUD for fills during the 90-day episode of care (shifting overlapping days forward) across all patients as well as just for those who had at least 1 fill within 14 days. For OUD-related clinical events, we captured the percentage of patients who had a drug overdose, inpatient detoxification or rehabilitation center stay, or an infection potentially secondary to injection drug use within 90 days of the index visit (eTable 3 in Supplement 1). For a full description of the outcomes, see eTable 5 in Supplement 1.

### Study Covariates

We captured age group (18-35, 36-50, 51-65, and ≥66 years), documented sex, and insurance type (commercial or Medicare Advantage) from enrollment data in the OptumLabs Data Warehouse. We defined rural-urban classifications using the rural-urban commuting area codes (metropolitan, micropolitan, small town, and rural),^29^ and county-level quartiles of race (percentage of population with White race) and poverty indicators (median household income) were identified from the US Census Bureau data.^30^ Race and ethnicity data were not available in the database. We used US Census Bureau data on county-level percentage of White residents because other race and ethnicity indicators at the county level were colinear with one another in regression modeling. We separated the counties into quartiles and linked them to patients according to the county listed in their claims. Additionally, we captured clinician specialty (primary care, psychiatry, registered nurse special service, anesthesiology, rehabilitation medicine, and other [neurology, pediatrics, and obstetrics and gynecology]).

### Statistical Analysis

We used χ^2^ tests to assess for bivariate differences between patients assigned to clinicians with low, medium, or high telemedicine use during the prepandemic and pandemic periods. To estimate the association between clinician telemedicine use and patient outcomes, we used a difference-in-differences approach.

For each outcome, we compared the changes in the prepandemic and pandemic periods between the patients seen by clinicians with low telemedicine use (ie, control group) and patients seen by clinicians with medium or high telemedicine use (ie, intervention groups). We compared the prepandemic and pandemic periods as 2 time points rather than as longitudinal rates per month or quarter to maximize statistical power for the smallest possible minimum detectable effect size for telemedicine. We estimated separate patient-level linear models (for visit rates and days’ supply) or logistic models (for binary outcomes, such as MOUD overdose) for each outcome, including indicators for telemedicine use group (high or medium vs low as reference), pandemic period, and an interaction term of the 2 variables, adjusting for clinician specialty and all patient characteristics. The key variable of interest in each difference-in-differences regression was the coefficient for the interaction term, which represented the differential change in each outcome that was attributable to clinician telemedicine use during the pandemic period. We clustered SEs at the clinician level.

Analyses were performed in SAS, version 9.4 (SAS Institute Inc). The 95% CIs of reported estimates reflected 0.025 in each tail or *P* ≤ .05, which indicated statistical significance. *P* values were 2-sided. In the sensitivity analysis, we repeated the main analyses and additionally required continuous enrollment in the behavioral health plan, but there was no difference in adjusted outcomes.

## Results

### Study Sample

The study sample contained 1768 clinicians who treated a total of 11 801 patients, including 5990 patients (50.8%) with an episode in the prepandemic period and 5811 (49.2%) with an episode in the pandemic period (Table 1 and Table 2). The mean (SD) patient age was 53.9 (15.7) years, and 5899 patients (50.0%) were female and 5902 (50.0%) were male (Table 1).

**Table 1.** Patient and Clinician Characteristics

Characteristic	No. (%)			Low vs medium *P* value^a^	Low vs high *P* value
	Clinician Telemedicine Use Group				
	Low	Medium	High		
Patients with OUD (N = 11 801)	4197 (35.6)	4308 (36.5)	3296 (27.9)	NA	NA
Clinicians (N = 1768)	589 (33.3)	590 (33.4)	589 (33.3)	NA	NA
**Patients**					
Age group, y					
18-35	699 (16.7)	551 (12.8)	608 (18.5)	<.001	.001
36-50	947 (22.6)	927 (21.5)	791 (24.0)		
51-65	1551 (37.0)	1559 (36.2)	1153 (35.0)		
≥66	1000 (23.8)	1271 (29.5)	744 (22.6)		
Documented sex					
Male	2204 (52.5)	2069 (48.0)	1629 (49.4)	<.001	.001
Female	1993 (47.5)	2239 (52.0)	1667 (50.6)		
Rurality					
Metropolitan	3376 (80.4)	3683 (85.5)	2897 (87.9)	<.001	<.001
Micropolitan	493 (11.8)	330 (7.7)	262 (8.0)		
Small town	229 (5.5)	205 (4.8)	89 (2.7)		
Rural	99 (2.4)	90 (2.1)	48 (1.5)		
Insurance type					
Commercial	2092 (49.9)	2060 (47.8)	1900 (57.7)	0.88	<.001
Medicare Advantage	2105 (50.2)	2248 (52.2)	1396 (42.4)		
Median household income in county					
1 (low)	1565 (37.3)	1305 (30.3)	677 (20.5)	<.001	<.001
2	1044 (24.9)	1080 (25.1)	879 (26.7)		
3	907 (21.6)	1078 (25.0)	872 (26.5)		
4 (high)	681 (16.2)	845 (19.6)	868 (26.3)		
% White population in county^b^					
1 (low)	449 (10.7)	517 (12.0)	416 (12.6)	<.001	<.001
2	1187 (28.3)	1328 (30.8)	1053 (32.0)		
3	1104 (26.3)	1346 (31.2)	901 (27.3)		
4 (high)	1457 (34.7)	1117 (25.9)	926 (28.1)		
**Clinicians**					
Specialty					
Primary care	286 (48.6)	303 (51.4)	156 (26.5)	<.001	<.001
Psychiatry	132 (22.4)	117 (19.8)	219 (37.2)		
RN special service	59 (10.0)	52 (8.8)	85 (14.4)		
Anesthesiology	61 (10.4)	78 (13.2)	85 (14.4)		
Rehabilitation medicine	34 (5.8)	34 (5.8)	37 (6.3)		
Other^c^	17 (2.9)	6 (1.0)	7 (1.2)		

Abbreviations: NA, not applicable; OUD, opioid use disorder; RN, registered nurse.

^a^ Unadjusted *P* values were estimated using χ^2^ tests.

^b^ Race and ethnicity data were not available in the OptumLabs Data Warehouse. US Census Bureau data on county-level percentage of White residents were used because other race and ethnicity indicators at the county level were colinear with one another in regression modeling.

^c^ Other specialties were neurology, pediatrics, and obstetrics and gynecology.

**Table 2.** Unadjusted OUD Visits and Outcomes by Clinician Telemedicine Use Group

Visit characteristic	Clinician telemedicine use group and period^a^					
	Low		Medium		High	
	Prepandemic^b^	Pandemic^c^	Prepandemic^b^	Pandemic^c^	Prepandemic^b^	Pandemic^c^
Patients with OUD, No./total No. (%)	2095/4197 (49.9)	2102/4197 (50.1)	2240/4308 (52.0)	2068/4308 (48.0)	1655/3296 (50.2)	1641/3296 (49.8)
OUD outpatient visits						
Total within 90 d after earliest visit, No.	6445	6848	5910	5442	4387	4424
Telemedicine, No. (%)	8 (0.1)	116 (1.7)	16 (0.3)	1032 (20.0)	83 (1.9)	2653 (60.0)
OUD visits per patient episode, mean (SD) No.^d^						
Total	3.1 (3.7)	3.3 (4.1)	2.6 (2.9)	2.6 (2.8)	2.6 (2.4)	2.7 (2.7)
In-person	3.1 (3.7)	3.2 (4.1)	2.6 (2.9)	2.1 (2.8)	2.5 (2.4)	1.1 (2.1)
Telemedicine	0 (0.1)	0.1 (0.3)	0 (0.1)	0.5 (1.0)	0.1 (0.5)	1.6 (2.0)
Proportion of patients with ≥1 MOUD fill, %						
Fill within 14 d	15.3	15.2	13.8	14.6	14.7	13.7
Fill within 90 d	19.1	18.5	18.0	18.1	19.9	17.5
Fill within 30-90 d among patients with a fill within 14 d	76.9	68.8	68.2	68.1	75.0	65.8
MOUD days’ supply among patients with ≥1 MOUD fill	20.2	20.5	20.8	20.9	18.8	18.2
Proportion of patients with OUD-related event within 90 d after index visit, %						
Overdose	2.3	2.1	1.7	1.9	1.9	1.8
Detoxification and rehabilitation	4.1	3.4	2.3	2.0	3.0	3.0
Injection-related infection	9.0	10.2	8.3	8.3	9.8	7.9
Any OUD-related event	14.7	14.6	11.4	11.7	14.0	12.1

Abbreviations: MOUD, medication for opioid use disorder; OUD, opioid use disorder.

^a^ The number of clinicians was 589 in the low, 590 in the medium, and 589 in the high telemedicine group.

^b^ Prepandemic period: March 14, 2019, to March 13, 2020.

^c^ Pandemic period: March 14, 2020, to March 13, 2021.

^d^ Refers to the total number of OUD visits a patient had in their 90-day episode.

Regardless of telemedicine use group, the highest proportion of patients were aged 51 to 65 years (37.0% in the low use and 35.0% in the high use groups). Patients seen by clinicians with high telemedicine use were less likely to reside in the lowest-income counties compared with those whose clinicians had low telemedicine use (20.5% vs 37.3%). Primary care physicians were more likely to be in the low vs high telemedicine use group (48.6% vs 26.5%), while psychiatrists were more likely to be in the high vs low use group (22.4% vs 37.2%) (Table 1).

### Outpatient and OUD Visits 

In the pandemic period, clinicians with low telemedicine use conducted a mean (SD) of 2.1% (2.5%) of all office visits (not just OUD visits) via telemedicine, while clinicians with high telemedicine use conducted a mean (SD) of 69.5% (18.6%) of their visits virtually (*P* < .001) (eTable 4 in Supplement 1). Clinician telemedicine use for OUD visits followed a similar pattern: 1.7% of visits were conducted virtually among those with low use vs 60.0% among those with high use for this subset of all visits (*P* < .001) (Table 2).

The mean number of OUD visits per episode remained stable in both the low and high telemedicine use groups over time (Figure). Patients seen by clinicians with low telemedicine use had a mean (SD) of 3.1 (3.7) OUD visits per patient episode in the prepandemic period and 3.3 (4.1) OUD visits per patient episode during the pandemic (Table 2). For clinicians with high telemedicine use, there was a mean (SD) of 2.6 (2.4) OUD visits per patient episode in the prepandemic period and 2.7 (2.7) visits in the pandemic period. In the adjusted model, there was no differential change in visit volume per patient from the prepandemic period to the pandemic period among clinicians with high vs low telemedicine use (differential change in OUD visit volume, –0.01; 95% CI, –0.28 to 0.26) (Table 3).

**Figure.**
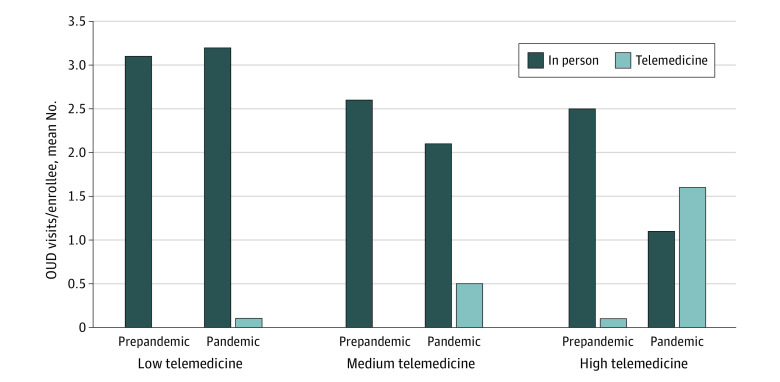
Unadjusted Mean Number of Opioid Use Disorder (OUD) Visits per Patient With OUD in a 12-Month Period The low, medium, and high telemedicine use groups were based on clinicians’ telemedicine use across all outpatient visits. The prepandemic period was March 14, 2019, to March 13, 2020, and the pandemic period was March 14, 2020, to March 13, 2021.

**Table 3.** Adjusted Differential Change in Continuous OUD Outcomes Among High and Medium vs Low Telemedicine Use Groups^a^

Continuous outcome	Medium vs low telemedicine use groups		High vs low telemedicine use groups	
	Differential change between the prepandemic and pandemic periods (95% CI)	*P* value	Differential change between the prepandemic and pandemic periods (95% CI)	*P* value
OUD visit volume				
Total	–0.13 (–0.42 to 0.16)	.38	–0.01 (–0.28 to 0.26)	.94
In person	–0.55 (–0.85 to –0.27)	.002	–1.51 (–1.79 to –1.24)	<.001
Telemedicine	0.43 (0.37 to 0.49)	<.001	1.51 (1.37 to 1.65)	<.001
MOUD days’ supply				
Among patients with ≥1 MOUD fill	0.29 (–1.09 to 1.68)	.68	–0.27 (–1.84 to 1.30)	.73

Abbreviations: MOUD, medication for opioid use disorder; OUD, opioid use disorder.

^a^ Continuous outcomes were modeled with linear regressions. Adjusted values in the table are the coefficient on the interaction term, representing the difference in outcomes between low vs medium and low vs high telemedicine use groups during the pandemic. Each statistical model was adjusted for age, documented sex, rurality, insurance type, median household income in county, racial demographics in county, clinician specialty, and state. We clustered SEs by state.

### MOUD Initiation and Retention

The proportion of patients seen by clinicians with low telemedicine use who initiated MOUD within 14 days of the index visit in the prepandemic and pandemic periods was 15.3% and 15.2%, respectively. The proportion of patients seen by clinicians in the high telemedicine use group were 14.7% in the prepandemic period and 13.7% in the pandemic period (adjusted odds ratio [OR], 1.00; 95% CI, 0.84-1.19) (Table 2 and Table 4). In the low telemedicine use vs high telemedicine use groups, patients who initiated MOUD within 14 days were equally likely to have at least 1 subsequent prescription in the 30 to 90 days after the index visit in the pandemic period (68.8% vs 65.8%; adjusted OR, 0.91 [95% CI, 0.74-1.12]) (Table 2 and Table 4). The mean (SD) days’ supply for patients with at least 1 fill within 90 days of the index visit was consistent across the prepandemic and pandemic periods for clinicians in both the low (20.2 [9.0] and 20.5 [9.1] days) and high (18.8 [9.5] and 18.2 [9.7] days) telemedicine use groups (differential change in days' supply, –0.27; 95% CI, –1.84 to 1.30).

**Table 4.** Adjusted Differential Change in Binary OUD Outcomes Among High and Medium vs Low Telemedicine Use Groups^a^

Binary outcome	Medium vs low telemedicine use groups		High vs low telemedicine use groups	
	aOR (95% CI)	*P* value	aOR (95% CI)	*P* value
MOUD initiation and supply				
≥1 MOUD within 14 d	1.01 (0.87-1.16)	.93	1.00 (0.84-1.19)	.96
≥1 MOUD within 90 d	1.00 (0.84-1.18)	.97	0.91 (0.74-1.12)	.38
≥1 MOUD fill within 30-90 d	0.79 (0.57-1.10)	.16	1.09 (0.74-1.60)	.67
Health care use within 90 d				
Overdose	1.12 (0.66-1.91)	.68	1.14 (0.72-1.83)	.58
Detoxification and rehabilitation	0.92 (0.45-1.88)	.82	0.84 (0.53-1.34)	.46
Injection-related infection	0.91 (0.71-1.16)	.44	0.81 (0.58-1.11)	.19
Any OUD-related event	1.01 (0.72-1.36)	.95	0.95 (0.73-1.24)	.69

Abbreviations: aOR, adjusted odds ratio; MOUD, medication for opioid use disorder; OUD, opioid use disorder.

^a^ Binary outcomes were modeled with logistic regressions. Adjusted values in the table are the coefficient on the interaction term, representing the difference in outcomes between low vs medium and low vs high telemedicine use groups during the pandemic. Each statistical model was adjusted for age, documented sex, rurality, insurance type, median household income in county, racial demographics in county, clinician specialty, and state. We clustered SEs by state.

### OUD-Related Events

The proportion of patients with at least 1 OUD-related clinical event was lower in the pandemic period compared with prepandemic period for those treated by clinicians in both the low (14.7% to 14.6%) and high (14.0% to 12.1%) telemedicine use groups; in adjusted analyses, there was no differential change between groups (adjusted OR, 1.01; 95% CI, 0.73-1.24) (Tables 2 and Table 4). There was also no difference between low and high telemedicine use groups when evaluating each OUD-related event individually. The proportion of patients with an overdose decreased from 2.3% to 2.1% in the low telemedicine use group and from 1.9% to 1.8% in the high telemedicine use groups (adjusted OR, 1.14; 95% CI, 0.72-1.83). Detoxification and rehabilitation admissions decreased from 4.1% in the prepandemic period to 3.4% in the pandemic period and remained at 3.0% in both periods in the high telemedicine use group (adjusted OR, 0.84; 95% CI, 0.53-1.34) (Table 2 and Table 4).

## Discussion

In a national sample of patients with OUD with commercial insurance or Medicare Advantage coverage, we found that treatment by clinicians with high telemedicine use was not associated with a different pattern of outpatient care or OUD-related events compared with treatment by clinicians with low telemedicine use. The total number of OUD visits per episode was consistent across the prepandemic and pandemic periods regardless of telemedicine uptake, suggesting that telemedicine almost entirely substituted, rather than supplemented, care. Overall, based on measures observable in claims data, telemedicine was comparable to in-person care, with no evidence of differential harm or benefit to patients who were seen by clinicians with high and medium vs low telemedicine use.

These results suggested that using telemedicine for OUD care was not associated with significantly lower rates of MOUD initiation or refills. These findings are consistent with those of prepandemic studies^31,32,33^ showing that buprenorphine delivered virtually had comparable patient retention and medication adherence to buprenorphine delivered in person. This study extends previous literature to the COVID-19 pandemic–era of telemedicine and its clinical implementation and suggests on a larger scale that telemedicine can safely be used to expand access to OUD care. While we were unable to observe visit appropriateness, the results of this study also do not suggest that telemedicine played a role in the increase in unnecessary or inefficient health care visits, an important concern raised by critics of telemedicine expansion.^34^

However, we did not find evidence of differential benefit either. Higher rate of telemedicine use was not associated with increased access, as measured by visit volume, given the consistent number of OUD visits across the prepandemic and pandemic periods. Greater telemedicine use was also not associated with increased MOUD initiation, refills, or days’ supply. The low rates of MOUD use both before and during the COVID-19 pandemic were consistent with rates reported in prior literature on access to OUD treatment among commercially insured populations.^35,36^ While telemedicine access may be part of a comprehensive policy package to promote MOUD access, there is substantial progress still needed to increase access, and telemedicine alone is unlikely to be sufficient.

It is important to note that patients who received OUD care from clinicians with high telemedicine use were concentrated in metropolitan counties with higher income and lower proportion of White residents. This finding could be consistent with concerns about a digital divide separating lower-income and rural areas in the US from mainstream technological advances that require broadband internet and other resources.^37,38,39^ In addition, given that high telemedicine use was not associated with changes in OUD care, it is possible that populations with access to clinicians with high telemedicine use had more resources to begin with compared with patients who had access to clinicians with low telemedicine use. Therefore, the digital divide may be a factor in the reduced potential of telemedicine to advance treatment access if additional measures are not taken to make telemedicine availability more equitable. High telemedicine use also was associated primarily with psychiatrists, aligning with previous reports of greater telemedicine use among behavioral health clinicians.^40^

### Limitations

This study has several limitations. It was an observational study; thus, we can report only associations and cannot provide conclusive evidence of any causal associations. However, we mitigated the selection bias that can occur in an observational study through the difference-in-differences design. Additionally, the findings may not be generalizable to other commercially insured populations, individuals enrolled in Medicaid and other Medicare programs, and individuals without insurance (a notable population since approximately one-fifth of adults with OUD are uninsured^41^). The outcomes capture only part of the complex process of access to care, and it is possible that telemedicine had benefits (or drawbacks) that we did not observe. While we were able to measure an individual’s receipt of MOUD, visit volume, and some OUD-related clinical events or adverse events, we were unable to measure receipt of long-acting buprenorphine implants and other important clinical outcomes, such as OUD relapse or patient functioning. In addition, the rates of overdose were limited to those who initiated OUD treatment and therefore were part of the cohort.

## Conclusions

In this cohort study, we found that after telemedicine expansion during the COVID-19 pandemic, patients with OUD experienced similar patterns of care and had similar outcomes whether they were treated by clinicians who predominantly used telemedicine or clinicians who provided in-person care. There was no evidence to suggest that telemedicine was unsafe or overused among clinicians with high vs low telemedicine use. Conversely, there was no evidence that telemedicine was associated with increased access or improved quality of care. The results of this study suggest that telemedicine is a comparable alternative for delivering care for OUD but not one that will substantially change care quality or access in the short term.

## Notes

Supplement 1. **eTable 1.** Facility Medications for Opioid Use Disorder (MOUD) **eTable 2.** Classification of Outpatient Visits **eTable 3.** Confirmatory Diagnosis CPT Codes **eTable 4.** Outpatient Visits (OUD and Non-OUD) Outcomes **eTable 5.** Outcome Descriptions **eFigure.** OUD Cohort Creation

Supplement 2.
Data Sharing Statement
