# Supplementary material for: Telemedicine Use and Quality of Opioid Use Disorder Treatment in the US During the COVID-19 Pandemic
Source: JAMA Netw Open. 2023 Jan 24;6(1):e2252381. doi: 10.1001/jamanetworkopen.2022.52381 (PMC10038015; doi:10.1001/jamanetworkopen.2022.52381)
Supplement: Supplement 1. — eTable 1. Facility Medications for Opioid Use Disorder (MOUD) eTable 2. Classification of Outpatient Visits eTable 3. Confirmatory Diagnosis CPT Codes eTable 4. Outpatient Visits (OUD and Non-OUD) Outcomes eTable 5. Outcome Descriptions eFigure. OUD Cohort Creation [file jamanetwopen-e2252381-s001.pdf]

Supplemental Online Content

Hailu R, Mehrotra A, Huskamp HA, Busch AB, Barnett ML. Telemedicine use and quality of opioid use disorder treatment in the US during the COVID-19 pandemic. *JAMA Netw Open*. 2023;6(1):e2252381. doi:10.1001/jamanetworkopen.2022.52381

eTable 1. Facility Medications for Opioid Use Disorder (MOUD)

eTable 2. Classification of Outpatient Visits

eTable 3. Confirmatory Diagnosis CPT Codes

eTable 4. Outpatient Visits (OUD and Non-OUD) Outcomes

eTable 5. Outcome Descriptions

eFigure. OUD Cohort Creation

This supplementary material has been provided by the authors to give readers additional information about their work.

© 2023 Hailu R et al. *JAMA Network Open.*

eTable 1. Facility Medications for Opioid Use Disorder (MOUD)

**Medication**	**HCPCS Codes**
Buprenorphine/naloxone	J0572, J0573, J0575
Long-acting injectable naltrexone	J2315
	HZ84ZZZ or HZ94ZZZ, but no AUD diagnosis (F10.x)
Buprenorphine without naloxone	J0571 (indicated for pregnant women receiving MOUD)

© 2023 Hailu R et al. *JAMA Network Open.*

eTable 2. Classification of Outpatient Visits

	**HCPCS Codes**
**Outpatient Codes**	90791-90792, 90832-90840, 90845-90849, 90853, 90857, 90865, 90867-90871, 90875-90876, 90880, 90900-90902, 90904, 90906, 90908, 90910, 97003, 97004, 98960-98962, 99058, 99078, 99201-99205, 99211-99215, 99241-99245, 99341-99345, 99347-99350, 99382-99387, 99392-99397, 99401-99404, 99408, 99409, 99411, 99412, 99420, 99441-99443, 99843, 99490, 99495, 99496, 99510, 0359T-0374T, G0155, G0175, G0351, G0396, G0397, G0438, G0439, G0442, G0443, G0463, G0466-G0470, G0505, G0507, G0513-G0515, G2025, G2067-G2080, G2086-G2088, H0001-H0007, H0014, H0016, H0022, H0023, H0028, H0029, H0031, H0034, H0036-H0050, H1011, H2000, H2001, H2010-H2033, H2037, H5010, H5020, H5025, H5030, H5220, H5230, H5240, H5299, M0064, S9454, S9482, S9484, S9485, T1006, T1007, T1011, T1012, T1015-T1018, T1023-T1027, T1040, T1041, T2010-T2015, T2018-T2024, T2036, T2037, Z0001, Z0002

We excluded all HCPCS codes that were specific for clinical settings outside of clinician offices (e.g., emergency departments, hospital inpatient, nursing home or dialysis facility codes). Below is the list of the HCPCS codes specific to outpatient care that were included in our analysis.

© 2023 Hailu R et al. *JAMA Network Open.*

eTable 3. Confirmatory Diagnosis CPT Codes

**Opioid Dependence**	F11.2
**Opioid Use or Abuse**	F11.1 F11.9
**Opioid Overdose**	T40.0X1, T40.0X2, T40.0X4, T40.0X5, T40.1X1, T40.1X2, T40.1X4, T40.2X1, T40.2X2, T40.2X4, T40.2X5, T40.3X1, T40.3X2, T40.3X4, T40.3X5, T40.4X1, T40.4X2, T40.4X4, T40.4X5
**Hepatitis C**	B17.1, B18.2, Z22.52
**Potentially Injection-related Infection**	
Phlebitis	I80.1-I80.9
Abscess and/or Cellulitis	G06.1, G06.2, L02.0-L02.4, L02.91, L03.0-L03.9, L98.3
Infectious Arthritis	M00.x
Infectious Endocarditis	B37.6, I33.0, I33.9, I38, I39
**Inpatient Detox/Rehabilitation Treatment Center**	
≥1 of Following Revenue International Classification of Diseases-10 Procedure Codes	
Revenue	0116, 0126, 0136, 0146, 0156, 0118, 0128, 0138, 0158, 1002
ICD-10	HZ.x
PLUS ≥ 1 of Following ICD-10 Diagnosis Codes	
ICD-10	F11.x

This table shows the list of codes for a confirmatory diagnosis or event — opioid overdose, hepatitis C (an infection potentially secondary to injection drug use), or an inpatient detox or rehabilitation treatment.

Codes adapted from:
Larochelle, M. R., Wakeman, S. E., Ameli, O., Chaisson, C. E., McPheeters, J. T., Crown, W. H., Azocar, F., & Sanghavi, D. M. (2020). Relative Cost Differences of Initial Treatment Strategies for Newly Diagnosed Opioid Use Disorder: A Cohort Study. Medical Care, 58(10), 919–926.

© 2023 Hailu R et al. *JAMA Network Open.*

eTable 4. Outpatient Visits (OUD and Non-OUD) Outcomes

Unadjusted outpatient utilization rates are listed below.

	**Provider Telemedicine Use Group**					
	**Low**		**Medium**		**High**	
	Pre*	Post**	Pre*	Post**	Pre*	Post**
Providers (n)	589	589	590	590	589	589
Enrollees with OUD (n)	2095	2102	2240	2068	1655	1641
**Outpatient Visits**						
Total In-Person	253,362	236244	268,710	210,237	204,612	66,602
Total Telemedicine	84	6578	582	53,853	3,113	155,041
Percent Telemedicine (%)	0.08	2.1	0.36	20.9	2.1	69.5
**Average Number of Outpatient Visits per Enrollee with OUD**						
Total	18.0	17.8	17.6	18.4	17.4	18.1
In-person	18.0	15.6	17.5	13.8	17.2	10.3
Telemedicine	0.0	2.2	0.1	4.5	0.2	7.9

*Pre: March 14th, 2019 – March 13th, 2020
**Post: March 14th, 2020 – March 13th, 2021

© 2023 Hailu R et al. *JAMA Network Open.*

eTable 5. Outcome Descriptions

**Outcome**	**Description**
OUD Outpatient Visits	The total number of OUD outpatient visits within the 90 days after the enrollees' index visit.
OUD Visit Volume	Across all OUD enrollees, the average number of outpatient visits each enrollee has throughout their 90-day episode
≥ 1 MOUD fill within 14 days	The percentage of enrollees who have at least 1 OUD outpatient visit and at least 1 MOUD fill within 14 days of their index visit
≥ 1 MOUD fill within 90 days	The percentage of enrollees who have at least 1 OUD outpatient visit and at least 1 MOUD fill within 90 days of their index visit
≥ 1 MOUD fill within 30-90 days among those with a fill within 14 days	The percentage of enrollees who have at least 1 more fill between the 30-90 days after their index visit, among those who have at least 1 MOUD fill within the 14 days of their index visit.
MOUD Days-Supply Among Those ≥ 1 MOUD fill	The average days-supply among enrollees with at least 1 MOUD fill
OUD-Related Event within 90 Days Following Index Visit (%)	The percentage of enrollees with an OUD-related event within the 90 days after their index visit. OUD related events included in this outcome are: overdose, detoxification/rehabilitation, injection-related infections, and the aggregate of these three.

© 2023 Hailu R et al. *JAMA Network Open.*

eFigure. OUD Cohort Creation

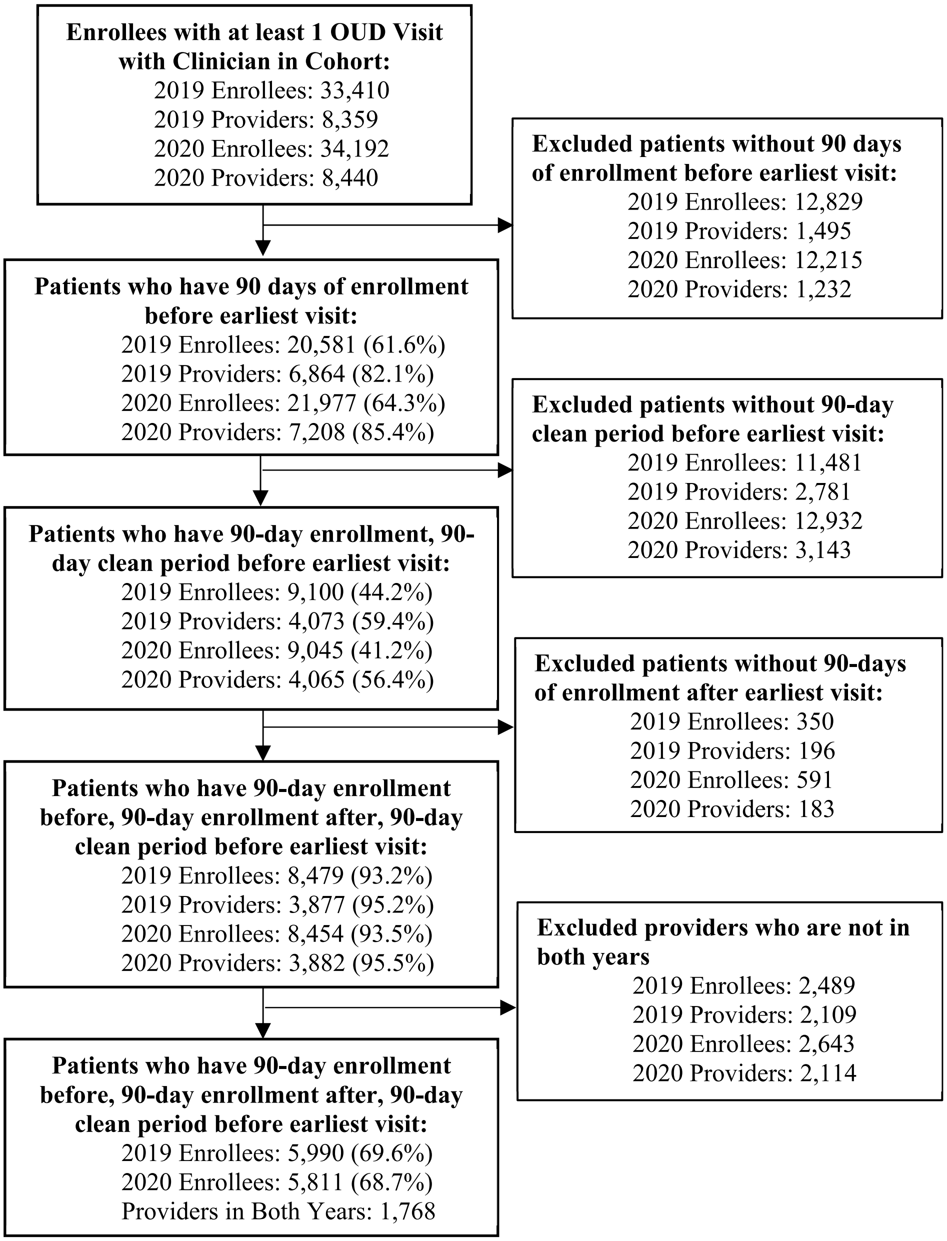

© 2023 Hailu R et al. *JAMA Network Open.*
