# Supplementary material for: Telemedicine Use and Quality of Opioid Use Disorder Treatment in the US During the COVID-19 Pandemic
Source: JAMA Netw Open. 2023 Jan 24;6(1):e2252381. doi: 10.1001/jamanetworkopen.2022.52381 (PMC10038015; doi:10.1001/jamanetworkopen.2022.52381)
Supplement: Supplement 2. — Data Sharing Statement [file jamanetwopen-e2252381-s002.pdf]

Data Sharing Statement

Hailu. Telemedicine Use and Quality of Opioid Use Disorder Treatment in the US During the COVID-19 Pandemic. *JAMA Netw Open*. Published January 24, 2023. doi:10.1001/jamanetworkopen.2022.52381

Data
Data available: No

Additional Information
Explanation for why data not available: The dataset used is proprietary, but available for qualified researchers via application.
